# The role of causal inference in health services research I: tasks in health services research

**DOI:** 10.1007/s00038-020-01333-2

**Published:** 2020-02-12

**Authors:** André Moser, Milo A. Puhan, Marcel Zwahlen

**Affiliations:** 1 Epidemiology, Biostatistics and Prevention Institute, University of Zurich, Hirschengraben 84, 8001 Zurich, Switzerland; 2 Institute of Social and Preventive Medicine, University of Bern, Mittelstrasse 43, 3012 Bern, Switzerland

## Introduction

In a recent issue of the American Journal of Public Health, Hernán and other colleagues strongly plea for causal thinking in scientific research where the research question investigates consequences of decisions and interventions (Ahern 2018; Begg and March 2018; Chiolero 2018; Glymour and Hamad 2018; Hernán 2018a, b; Jones and Schooling 2018). Hernán argues that causal reasoning improves quality of observational research; however, the causal terminology is often loomed by the ‘association is not causation’ argument and is viewed with skepticism (Hernán 2018b). Health services research (HSR) supports decision making by investigating the effect of complex ‘interventions’ or ‘policies’ on different healthcare system outcomes (Glass et al. 2013). Thus, some of the research questions in HSR are inherently causal. Surprisingly, there is no consensus on how to integrate causal inference into tasks of HSR (Dowd 2011; O’Malley 2011; Pearl 2011; Hernán et al. 2019). Typically, tasks in data science are classified into ‘description’, ‘modeling’ and ‘causal inference’ (Hernán et al. 2019). In the present *Hints and Kinks,* we explain why a solidly principled causal inference framework should be integrated into the tasks of HSR.

### Tasks in health services research

Table 1 shows three examples of healthcare system interventions (HSIs). We use those examples to highlight the differences between the ‘core’ tasks in HSR.

**Table 1:** Examples of healthcare system interventions, by health services research tasks

Intervention or campaign	Health services research task		
	Description*	Modeling*	Causal inference*
Outpatient before inpatient treatment	How many arthroscopic meniscectomies were performed last year?	Which healthcare system predictors are related with surgical site infections after arthroscopic meniscectomies?	Does a shift from inpatient to outpatient treated arthroscopic meniscectomies increase the risk for surgical site infections after an arthroscopic meniscectomy?
Task shifting	What are observed rates of post-surgical treatment and patient outcomes, by different skill grades?	Which skill-grade-related predictors are associated with improved patient outcomes?	Does task shifting reduce resources and improve patient outcomes?
Hospital merger	What are the observed hospital readmission rates?	What is the probability of a hospital readmission after a hospital merger?	Does a hospital merger reduce readmission rates?

*Task ‘description’: Describes the study population and informs stakeholders, policy makers and the public about the current situation of a healthcare system. Task ‘modeling’: Quantifies associations of an outcome with certain characteristics of the study population or the healthcare system and allows for predictions of future events. Task ‘causal inference’: Investigates the impact of an intervention or policy under different hypothetical scenarios (‘what-if’ questions)

The first example in Table 1 mentions the shift from inpatient to outpatient care, which has been successfully implemented in many low- and high-income countries (Yuan et al. 2017; Vogenberg and Santilli 2018; Bhatt and Bathija 2018). Switzerland, for example, published a list of mandatory outpatient treatments and surgeries in 2019 (Swiss Federal Office of Public Health 2019). As outpatient surgeries come with lower (official) reimbursement values, this shift will result automatically in lower reimbursement costs. But it might come with more complications such as surgical site infections which might add downstream costs. Such a HSI requires that involved stakeholders (for example, hospitals and health insurers), policy makers (for example, the government and regulators) and the public (i.e., patients and medical personnel) are informed about the status quo of the current healthcare system–in this example—of selected inpatient and outpatient treatments for a comparison. Obviously, they need a ‘description’ of population outcomes, say, rates of meniscectomy procedures. The ‘description’ of a study population and population outcomes is unquestioned a main task in HSR.

The second example in Table 1 is about task shifting, i.e., the shift of selected healthcare tasks among healthcare professionals. For example, nurses perform certain post-surgical treatments more efficiently than medical doctors. Task shifting has been implemented in low-, middle- and high-income countries worldwide (Ogedegbe et al. 2014; Seidman and Atun 2017; Orkin et al. 2019). Involved aspects of HSR in this HSI include the role of medical personnel and patients in shared decision making processes, hospitals as policy makers which implement work policies, but also authorities for ensuring patient safety and quality of treatments. Besides a ‘description’ of how often healthcare tasks are performed by different medical personnel, it is also of interest which predictors are associated (say, skill-grade) with a specific healthcare outcome (say, length of hospital stay). In brief, ‘description’ summarizes the study setting, whereas ‘modeling’ quantifies associations among a study outcome and certain characteristics to make predictions for other–possibly future—settings (Hernán et al. 2019). There is no doubt that to ‘describe’ and to ‘model’ are important tasks in HSR. But, obviously, answering the questions in the first two columns of Table 1 only in part supports decision making—a process of actions or ‘doing’ (Pearl 2009)—which is ultimately linked to a causal ‘what-if’ question: What is the effect of an intervention when everything happened under a situation, say A, versus when everything happened under a situation B. Let us consider the third example in Table 1, which focuses on hospital mergers, for a better explanation. Over the past years, consolidation efforts between hospitals have been implemented in many countries to optimize patient treatments, quality outcomes and economic outcomes (Kristensen et al. 2010; Hayford 2012; Giancotti et al. 2017). Let us consider in the following the situation that two hospitals merged. A natural question for stakeholders (i.e., a hospital group) and policy makers (i.e., health system authorities and regulators) is then: What would have happened to the outcome of quality indicators (say, hospital readmissions) when the merger would not have been implemented? For a hospital group this is a key question to justify a consolidation effort, whereas for authorities and regulators an answer to this question is needed for health system planning and evaluation, but actually is not available when the decisions need to be taken. Importantly, such a question moves away from ‘observing’ or ‘modeling’ to a situation of ‘understanding’ the effect of an action, i.e., what is the effect of the hospital merger on quality outcomes? This is a causal question and can often not be answered by associational effect summaries from ‘descriptive’ or ‘modeling’ approaches.

### The need for a causal inference framework in health services research

Unfortunately, public health decisions on interventions or policies are often only based on ‘descriptive’ and ‘modeled’ results, without the integration of a solidly principled causal inference framework. Despite both, ‘traditional’ and ‘causal inference’, approaches have their proper legitimation in the investigation of specific research questions, many researchers and students in the field of HSR are not trained to the notions of causal inference. Modern causal inference identifies effects of interventions by using the concept of counterfactuals, which are a key component of a causal inference framework (Hernán 2004; Glass et al. 2013; Zwahlen and Salanti 2018). A counterfactual outcome is an outcome which is ‘counter to the fact’, that is, a hypothetical outcome which is actually not observed (Rubin 1974; Hernán 2004). In the example of the above hospital merger, one likes to compare the actual—under the situation of the hospital merger–observed hospital readmissions with hospital readmissions when the hospital merger *would not have been* implemented, i.e., outcomes which are actually never observed. Counterfactuals allow to mathematically define a causal effect, which is conceptually different from an associational effect. In a follow-up *Hint and Kinks,* we continue on the introduction of a principled framework for causal inference in HSR (Moser et al. 2020).

## Discussion

As Hernán and others, we believe that causal reasoning plays an important role in HSR to support public health decision making. This can only be done by using an explicit ‘causal inference’ framework, besides the traditional tasks of ‘description’ and ‘modeling’. None of these tasks should be viewed superior to another, but each should be adequately chosen, related to the intended research question (Hernán et al. 2019). Unfortunately, the concept of causal inference is not a regular part in the curriculum of researchers (Dowd 2011; Glass et al. 2013; Begg and March 2018; Chiolero 2018). We plea for an integration of causal concepts in the education of health services researchers, epidemiologists, public health practitioners, and other related professions, to foster future HSR.
